# Associations of Parenting Style and Resilience With Depression and Anxiety Symptoms in Chinese Middle School Students

**DOI:** 10.3389/fpsyg.2022.897339

**Published:** 2022-07-01

**Authors:** Zhihai Qiu, Ying Guo, Jun Wang, Hongbo Zhang

**Affiliations:** ^1^The School of Mental Health and Psychological Sciences, Anhui Medical University, Hefei, China; ^2^Psychological Center of Hefei No.1 High Senser School, Hefei, China; ^3^Department of Maternal, Child and Adolescent Health, School of Public Health, Anhui Medical University, Hefei, China; ^4^MOE Key Laboratory of Population Health Across Life Cycle, Anhui Provincial Key Laboratory of Population Health and Aristogenics, Hefei, China

**Keywords:** parenting style, resilience, depression symptoms, anxiety symptoms, latent profile analysis (LPA)

## Abstract

**Background:**

Parenting style and resilience are independently associated with symptoms of depression and anxiety. However, no study has tested the interaction effects between the patterns of parenting style and resilience on mental health in adolescent populations. Therefore, this study aimed to explore the interaction effects between the patterns of parenting style and resilience on depression/anxiety symptoms among middle school students in China.

**Methods:**

A sample of 2,179 Chinese middle school students were included in this study. Latent profile analysis (LPA) was used to examine parenting style patterns. Multivariable logistic regression was used to analyze the associations of different parenting patterns and resilience with depression/anxiety symptoms, as well as the interaction effect.

**Results:**

Latent profile analysis results showed that the most suitable model included three-profile solution, which were labeled as positive parenting, negative parenting, and moderate parenting. Subsequent analyses indicated that students across profiles exhibited significant differences in their depression/anxiety symptoms. Specifically, compared to moderate parenting, negative parenting was positively associated with depression/anxiety symptoms, while positive parenting was negatively associated with these symptoms. Moreover, low levels of resilience were positively associated with depression/anxiety symptoms compared to a high level of resilience. Although the interaction effect was not significant, there were differences in the associations between different parenting patterns and symptoms of depression and anxiety when stratifying resilience.

**Conclusion:**

The present study identified three-profile solution of parenting styles among Chinese middle school students using LPA as a person-centered approach. Future interventions targeting depression/anxiety symptoms in adolescents may consider the potential influence of patterns of parenting styles, or improved resilience, to achieve better intervention outcomes.

## Introduction

Depression and anxiety are the most common psychopathological symptoms in adolescents. Depression symptoms manifest as persistent sadness, loss of interest, fear of the future, and potential suicidality (Naab et al., 2015; Wartberg et al., 2018). Anxiety is an emotion characterized by feelings of tension, worried thoughts, and physical changes such as increased blood pressure (Organization, 2013). While adolescents are in the transition period from childhood to adulthood, simultaneously experiencing great changes in social roles and living environment means they are more prone to depression, anxiety, and other adverse emotions. The global prevalence of clinically elevated depressive/anxiety symptoms among adolescents was approximately 25.2 and 20.5%, respectively. Furthermore, a survey completed by Chinese high school students from 21 provinces and autonomous regions revealed that the prevalence of depressive and anxiety symptoms was 43.7 and 37.4% among students (Zhou et al., 2020).

Parenting style refers to the attitude, goals, and emotional atmosphere that parents use to raise and educate their children, which remain relatively stable in different situations (Darling and Steinberg, 1993). Parental warmth and strictness have been identified as the two main independent dimensions of parenting style. From these, four parenting styles were identified: authoritative (marked by high warmth and high strictness), authoritarian (marked by low warmth and high strictness), indulgent (marked by high warmth and low strictness), and neglectful (marked by low warmth and low strictness) parenting (Martinez et al., 2020). A systematic literature review showed that (Gorostiaga et al., 2019) parental warmth, behavioral control, and autonomy granting were inversely correlated with depressive symptoms in adolescents, while parental psychological control and severe control were positively related to depression/anxiety symptoms. Romero-Acosta et al. (2021) found that compared with those who experienced authoritative parenting, students who experienced neglectful parenting styles generally had lower symptoms of anxiety. Prior studies on parenting style have mainly used variable-centered methods, which makes it difficult to examine how the various factors that constitute parenting style combine within individuals and affect children. In this regard, it is necessary to examine parenting style by using latent profile analysis (LPA) with a person-centered approach. A person-centerd approach can provide more insight than a variable-centerd approach into the parenting style of specific populations with heterogeneous characteristics (Lee and Han, 2021).

In addition to the influence of parenting style on depressive and anxiety symptoms in adolescents, resilience has also been suggested as influential. For instance, previous research has confirmed that higher levels of resilience are related to lower levels of depressive symptoms in children and adolescents (Wingo et al., 2010; Wu et al., 2017; Lee et al., 2021). Furthermore, a three-wave cross-lagged design indicated (Lau, 2022) an unstable reciprocal correlation between resilience and depression over time and a stable reciprocal correlation between resilience and anxiety symptoms.

According to the existing mental resilience model (Mandleco and Peery, 2000), the factors affecting mental resilience can be divided into internal factors and external factors. Internal factors are biological factors (e.g., genetic) and psychological factors (e.g., optimism and self-esteem), while external factors refer to factors inside and outside the family (e.g., family functions and social support). Among them, parenting style is an important external factor. And adolescent mental health can be affected by parenting style (Milevsky et al., 2007). Research on resilience and parenting style has shown that authoritative parenting has a significantly positive impact on resilience (Kritzas and Grobler, 2005; Zakeri et al., 2010). Therefore, based on previous surveys, the following three hypotheses were proposed: (1) exploring different patterns of parenting style by LPA; (2) parenting style and resilience are independently correlated with anxiety and depressive symptoms in Chinese adolescents; and (3) there are interaction effects of parenting style and resilience on depressive and anxiety symptoms.

## Materials and Methods

### Participants

This was a cross-sectional study, and a cluster sampling design was used to select the sample population from Hefei City, Anhui Province during the period of September to October 2021. A total of 2,936 senior high school students were recruited for this study and 2,879 valid questionnaires were received (57 questionnaires with missing values >15% were deleted). The efficacy rate was 98.06%. Of them, the mean age was 16.7 ± 1.8 years, 63.4% were boys (1,319) and 36.6% were girls (760).

This study was approved by the Ethics Committee of Anhui Medical University. Informed consent was obtained from all students, parents, and teachers before the survey.

### Measures

#### Sociodemographic Data

Sociodemographic data were collected using an anonymous questionnaire, which included gender, only child status, registered residence, self-reported academic performance (poor, medium, or good), and parents’ education level.

#### Parenting Styles

The Egma Minnen av Bardndosnauppforstran (EMBU) standard edition, which was co-edited by Perris et al. (1980) of the Psychiatry Department at Umea University in Sweden, was used to evaluate parenting attitudes and behaviors. The Chinese version of the EMBU, which was first introduced in 1993 (Li et al., 2012). Paternal and maternal parenting styles comprise 58 and 57 items, respectively. It includes six subscales: parental emotional warmth, parental rejection and denial, parental severe punishment, parental excessive interference, parental favoring subjects, and paternal over-protection. In this study, only child accounted for 44.3% (921), so the subscale of parental favoring subjects were excluded. Furthermore, except for paternal over-protection, the other four sub-scales all include two dimensions of father and mother, respectively. In summary, there are nine dimensions. Each item was adopted a 4-point Likert scale ranged from 1 (never) to 4 (most of time), and entries were added according to different dimensions. In this study, the Cronbach’s α coefficients for paternal and maternal parenting styles were 0.813 and 0.807, respectively.

#### Depression Symptoms

The Center for Epidemiological Studies Depression Scale (CES-D) is a widely used depressive symptom screening tool worldwide (Radloff, 1977). Compared with other depression scales, this scale focuses more on an individual’s emotional experiences and less on the somatic symptoms of depression. The scale has a total of 20 items, and each item is rated on a 4-point Likert scale ranging from 0 (rarely or none), 1 (some or a little), 2 (occasional or moderate), and 3 (most or all of the time), of which four items on positive affect were reverse-scored. Adolescents with depressive symptoms were classified based on whether their total score was ≥20 (Huang et al., 2021; Sun et al., 2021). The overall Cronbach’s alpha coefficient for the scale was 0.910.

#### Anxiety Symptoms

The Self-rating Anxiety Scale (SAS) is a 20-item retrospective self-report questionnaire designed to detect symptoms related to anxiety in the general population (Zung, 1971). Responses to each question range from 1 (no or little time at all) to 4 (most or all of the time), equating to a total scale raw score of 20–80, then conversed to a index score with a potential range of 25–100. The index score is “derived by dividing the sum of the values (raw scores) obtained on the 20 items by the maximum possible score of 80, converted to a decimal and multiplied by 100” (Zung, 1971). The higher the standard score, the more severe the symptom. Subsequently, 50 points were set as the cut-off standard for anxiety symptoms (Zung, 1971; Huang et al., 2021). In this study, Cronbach’s α coefficient was 0.799.

#### Resilience

Resilience was measured using the Self-rating Resilience Scale for Middle School Students (SRSMSS) compiled by Hu et al. (2011). A total of 26 items were used, including six dimensions: problem solving, cooperation and communication, self-efficacy, goals and aspirations, self-awareness, and empathy. According to the situation experienced by participants over the previous two weeks, each option was scored on a scale of 1–5, indicating “never,” “occasionally,” “sometimes,” “often,” and “always”. Students in this study were categorized as having low or high resilience, with P75 as the cutoff point. The SRSMSS has high reliability and validity (Lü et al., 2013), and Cronbach’s α coefficient was 0.931 in the present study.

### Statistical Analysis

SPSS 23.0 was used for data processing and analysis, and the inspection level was α = 0.05. First, we used χ^2^ tests to compare the associations between sociodemographic variables and anxiety and depression symptoms. Second, in order to identify parenting styles of different clustering patterns, Mplus version 7.4 was utilized for LPA. Indicators for the primary analysis included nine dimensions from the EMBU. The number of latent classes was determined based on the commonly used fit statistics of Akaike’s Information Criterion (AIC), Bayesian Information Criterion (BIC), and sample-size adjusted BIC (a-BIC); lower numbers indicated better model fit, as well as the bootstrapped likelihood ratio test (BLRT), which is a significance test for model improvement with the addition of each potential class. Generally, a relative entropy >0.7 indicates that the model is in the acceptable range, and the proportion of each classification group should be >5% of the total population. Third, multivariable logistic regression was used to analyze the associations of parenting style and resilience with depressive and anxiety symptoms, controlling for gender, only child status, registered residence, self-reported academic performance, and parents’ education level. In examining the association of resilience with depressive and anxiety symptoms, we also used the thresholds of resilience score ≥P_67_ and ≥P_90_ for sensitivity analysis. Binary logistic regression was used to analyze the relationship between the interaction of parenting style and resilience with depressive and anxiety symptoms. Finally, a stratified analysis of the relationship between parenting style and anxiety as it relates to depression symptoms was conducted according to the level of resilience.

## Results

### Characteristics of Participants

As shown in Table 1, of 2,079 participants, 63.4% were boys and the rates of depression/anxiety symptoms were 26.0% (541) and 13.4% (279), respectively. Depression/anxiety symptoms were more common in girls and in rural areas (*P* < 0.001). Rates were highest among students with poor self-reported academic performance (*P* < 0.001). No statistically significant differences in depression/anxiety symptoms were found in students who were an “only child” or in fathers’ educational level (*P* > 0.05).

**TABLE 1:** Characteristics of participants by depression/anxiety symptoms.

Variable	Total (*N* = 2079)	Depression symptoms *n*(%)			Anxiety symptoms *n*(%)		
		No	Yes	χ*^2^*	No	Yes	χ*^2^*
**Gender**				5.33*			17.16**
Male	1319 (63.4)	998 (75.7)	321 (24.3)		1173 (88.9)	146 (11.1)	
Female	760 (36.6)	540 (71.1)	220 (28.9)		627 (82.5)	133 (17.5)	
**Only child**				0.55			0.11
Yes	921 (44.3)	674 (73.2)	247 (26.8)		800 (86.9)	121 (13.1)	
No	1158 (55.7)	864 (74.6)	294 (25.4)		1000 (86.4)	158 (13.6)	
**Registered residence**				13.77**			11.06**
Rural	825 (39.7)	574 (69.6)	251 (30.4)		689 (83.5)	136 (16.5)	
Urban	1254 (60.3)	964 (76.9)	290 (23.1)		1111 (88.6)	143 (11.4)	
**Self-reported academic performance**				12.88*			11.55*
Good	228 (11.0)	163 (71.5)	65 (28.5)		191 (83.8)	37 (16.2)	
Medium	1731 (83.3)	1302 (75.2)	429 (24.8)		1516 (87.6)	215 (12.4)	
Poor	120 (5.8)	73 (60.8)	47 (39.2)		93 (77.5)	27 (22.5)	
**Father’s education level**				3.11			4.13
Primary or below	85 (4.1)	60 (70.6)	25 (29.4)		70 (82.4)	15 (17.6)	
Junior middle school	599 (28.8)	431 (72.0)	168 (28.0)		512 (85.5)	87 (14.5)	
Senior middle school	515 (24.8)	381 (74.0)	134 (26.0)		442 (85.8)	73 (14.2)	
College or above	880 (42.3)	666 (75.7)	214 (24.3)		776 (88.2)	104 (11.8)	
**Mother’s education level**				10.44*			3.32
Primary or below	188 (9.0)	145 (77.1)	43 (22.9)		165 (87.8)	23 (12.2)	
Junior middle school	655 (31.5)	455 (69.5)	200 (30.5)		554 (84.6)	101 (15.4)	
Senior middle school	524 (25.2)	401 (76.5)	123 (23.5)		459 (87.6)	65 (12.4)	
College or above	712 (34.2)	537 (75.4)	175 (24.6)		622 (87.4)	90 (12.6)	

*P < 0.05. **P < 0.001.

### Latent Profile Analysis of Parenting Styles

Models with one to five profiles were tested in the LPA. The three-profile solution was regarded as the most suitable based on the indices (Table 2), which showed the high entropy (0.896), *p*-values of LMR and BLRT test were significant. In addition, the average posterior class membership probability scores were acceptable among the groups (0.929–0.966; Supplementary Table 1). Due to the lower AIC, BIC, and aBIC for the four-profile solution, we also compared and analyzed it (Supplementary Figure 1). The results showed that there were not much distinction between profiles solution. Moreover, the proportion of a profile group was 4.0% (<5%) of the total population, it did not meet the selection requirements of the model. Meanwhile, further stratified analysis showed that the sample size of some groups was too small to obtain effective results. Therefore, based on the above reasons and the principle of model simplicity, we chose the three-profile solution for the suitable of in the present study.

**TABLE 2:** Fitting information was analyzed by latent categories of parenting styles.

Profiles	*df*	*AIC*	*BIC*	*aBIC*	*Entropy*	*LMR*	*BLRT*	Profile probability
1	18	115,441.750	115,543.263	–	–	–	–	–
2	28	109,155.840	109,313.750	109,224.791	0.926	<0.001	<0.001	74.80/25.20
3	38	107,232.838	107,447.165	107,326.435	0.896	0.041	<0.001	58.87/31.99/9.22
4	48	106,382.317	106,653.019	106,500.519	0.888	0.022	<0.001	30.54/12.17/53.3/3.99
5	58	105,739.617	106,066.716	105,882.445	0.849	0.344	<0.001	41.08/18.33/16.26/20.30/4.04

*df, degrees of freedom; AIC, Akaike Information Criteria; BIC, Bayesian Information Criteria; aBIC, Adjusted Bayesian Information Criteria; LMR, Lo-Mendell-Rubin Likelihood Ratio; BLRT, Bootstrapped Likelihood Ratio Tests.*

**FIGURE 1:**
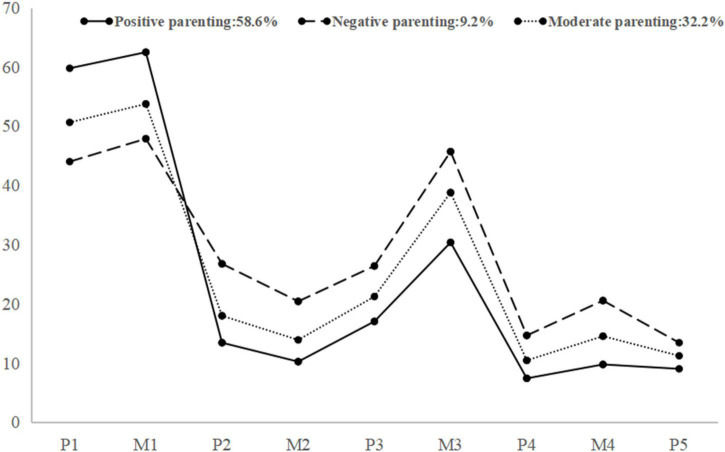
Plot of 3 latent profiles of parenting styles. P1 = paternal emotional warmth; M1 = maternal emotional warmth; P2 = paternal severe punishment; M2 = maternal severe punishment; P3 = paternal excessive interference; M3 = maternal excessive interference; P4 = paternal rejection and denial; M4 = maternal rejection and denial; P5 = paternal over protection.

Figure 1 shows the three parenting profiles. profile 1 showed a high level of parental emotional warmth and was labeled as “positive parenting” (58.6%). In contrast, profile 2 was characterized by a high probability of severe punishment, excessive interference, rejection and denial, and overprotection, thus we labeled it as “negative parenting” (9.2%). Meanwhile, profile 3 consisted of a moderate probability across nine dimensions of parenting styles, which was labeled as “moderate parenting” (32.2%).

### Patterns of Parenting Styles and Resilience With Depression/Anxiety Symptoms

Table 3 shows the association of the three profiles of parenting styles and resilience with depression/anxiety symptoms. After adjusting for sociodemographic characteristics (gender, only child status, registered residence, self-reported academic performance, and parents’ education level), compared with the “moderate parenting” pattern, logistics regression analysis showed that the “negative parenting” pattern was positively associated with depression symptoms (OR = 1.82, 95%CI: 1.30–2.53) and anxiety symptoms (OR = 2.01, 95%CI: 1.38–2.92). Conversely, the “positive parenting” pattern was negatively associated with depression symptoms (OR = 0.30, 95%CI: 0.24–0.37) and anxiety symptoms (OR = 0.32, 95%CI: 0.24–0.43). With a high level of resilience (≥P_75_) as the control group, a low level of resilience was positively associated with depression (OR = 6.74, 95%CI: 4.73–9.61) and anxiety symptoms (OR = 2.80, 95%CI: 1.92–4.09). Across different gender subgroups, the above association results were similar (Supplementary Tables 2, 3). Using the thresholds of resilience score ≥P_67_ and ≥P_90_ for separate data analysis, we found that the associations of different level of resilience and depression/anxiety symptoms (Supplementary Table 4) were similar to those found with ≥P_75_ (Table 3).

**TABLE 3:** Association of parenting style and resilience with depression/anxiety symptoms in adolescents.

Variable	Total (*N* = 2079)	Depression symptoms			Anxiety symptoms		
		*n*(%)	Crude *OR* (95% *CI*)	Adjusted *OR* (95% *CI*)^a^	*n*(%)	Crude *OR* (95% *CI*)	Adjusted *OR* (95% *CI*)^a^
***Parenting style***							
Positive parenting	1,224 (58.9)	194 (15.8)	0.32 (0.25–0.39)**	0.30 (0.24–0.37)**	91 (7.4)	0.34 (0.25–0.45)**	0.32 (0.24–0.43)**
Negative parenting	190 (9.1)	98 (51.6)	1.78 (1.29–2.46)*	1.82 (1.30–2.53)**	60 (31.6)	1.94 (1.35–2.78)**	2.01 (1.38–2.92)**
Moderate parenting	665 (32.0)	249 (37.4)	1.00	1.00	128 (19.2)	1.00	1.00
***Resilience***							
Low	1549 (74.5)	504 (32.5)	6.43 (4.53–9.13)**	6.74 (4.78–9.61)**	245 (15.8)	2.74 (1.88–3.98)**	2.80 (1.92–4.09)**
High	530 (22.5)	37 (7.0)	1.00	1.00	34 (6.4)	1.00	1.00

^a^Adjusted for gender, only child, registered residence, self-reported academic performance, parents’ education level. *P < 0.05. *P < 0.001.

The patterns of parenting style and resilience were highly correlated in the present study (Supplementary Table 5). However, there were no interaction effects between parenting style and resilience on depression/anxiety symptoms in the total sample (Supplementary Table 6). Table 4 shows the stratified data analysis by resilience group level. A significant association of depression symptoms (C1: OR = 0.34, 95%CI: 0.27–0.43; C2: OR = 1.79, 95%CI: 1.25–2.58) and anxiety symptoms (C1: OR = 0.36, 95%CI: 0.26–0.50; C2: OR = 1.96, 95%CI: 1.32–2.92) with the patterns of parenting styles was seen in adolescents with low levels of resilience. At a high level of resilience, the “positive parenting” pattern was negatively associated with depressive symptoms (OR = 0.39, 95%CI: 0.18–0.83) and anxiety symptoms (OR = 0.28, 95%CI: 0.13–0.62), and there was no significant association of “negative parenting” patterns with depressive symptoms and anxiety symptoms.

**TABLE 4:** Association of parenting style with depression/anxiety symptoms by different level of resilience.

Parenting style	Depression symptoms			Anxiety symptoms		
	*n*(%)	Crude OR (95% CI)	Adjusted OR (95% CI)^a^	*n*(%)	Crude OR (95% CI)	Adjusted OR (95% CI)^a^
**Low**						
Positive parenting	175 (21.0)	0.36 (0.28–0.45)**	0.34 (0.27–0.43)**	75 (9.0)	0.38 (0.27–0.51)**	0.36 (0.26–0.50)**
Negative parenting	93 (57.1)	1.78 (1.25–2.53)*	1.79 (1.25–2.58)*	55 (33.7)	1.94 (1.32–2.84)*	1.96 (1.32–2.92)*
Moderate parenting	236 (42.8)	1.00	1.00	115 (20.8)	1.00	1.00
**High**						
Positive parenting	19 (4.9)	0.39 (0.19–0.83)*	0.39 (0.18–0.83)*	16 (4.1)	0.33 (0.15–0.71)*	0.28 (0.13–0.62)*
Negative parenting	5 (18.5)	1.75 (0.57–5.41)	1.75 (0.53–5.72)	5 (18.5)	1.75 (0.57–5.41)	1.99 (0.60–6.61)
Moderate parenting	13 (11.5)	1.00	1.00	13 (11.5)	1.00	1.00

^a^Adjusted for gender, only child, registered residence, self-reported academic performance, parents’ education level. *P < 0.05. **P < 0.001.

## Discussion

This study examined the effects of parenting style patterns and resilience on depression/anxiety symptoms among adolescents. Results revealed that diverse profilees of parenting styles with different resilience levels have various relationships with depression/anxiety. However, we found no interaction between parenting style, resilience, depression, and anxiety symptoms among adolescents.

In the current study, we found that the prevalence of depression/anxiety symptoms among adolescents was 26.0 and 13.4%, respectively, which was lower than that reported by Zhou et al. (2020) (43.7 and 37.4%, respectively). Severval studies indicated that the prevalence of depressive/anxiety symptoms among adolescents in China may be higher than in other countries (Denda et al., 2006; Liu and Lu, 2012; Polanczyk et al., 2015; Murshid, 2017). It may be explained by the following reasons. First, Chinese teenagers are burdened with homework, high academic pressure and little physical activity, which may increase the risk of depressive/anxiety symptoms (Tepper et al., 2008). Second, the difference in prevalence of depression/anxiety symptoms may be associated with the different scales, evaluation criteria, and the different ages of the study populations (Wang et al., 2016; Rao et al., 2019). Third, the level of economic development in different regions, interpersonal relationships, and specific cultural factors in different regions could have contributed to the difference in results (Tang et al., 2019; Wang S. et al., 2019; Wang Y. Y. et al., 2019).

In the real world, parenting styles are not limited to a single form, and each parenting style is an integrated combination of behaviors. Thus, in this study, LPA was used to classify parenting styles into three profiles: “positive parenting” (58.6%), “negative parenting” (9.2%) and “moderate parenting” (32.2%). Similarly, a Chinese study conducted by Wu et al. (2016) labeled parenting styles as “positive parenting,” “negative parenting,” and “mixed parenting”. These results differed from the comprehensive model of parenting styles used in other studies. For example, Ayón et al. (2015) investigated different parenting styles and labeled four profiles as “family parenting,” “child-centered parenting,” “moderate parenting,” and “disciplinarian parenting.” The reason for the difference in results may be that these studies were conducted in different cultural contexts and used different parenting style questionnaires. In summary, parenting styles may not be limited to one form, which suggests that different cultural backgrounds and different questionnaires for the comprehensive model of parenting styles can be further explored.

This study revealed that, compared with the “moderate parenting” profile, the “positive parenting” and the “negative parenting” profiles were both related to depression/anxiety symptoms among adolescents, while positive parenting was a protective factor for depression/anxiety. Wang et al. (2021) identified four parenting styles through LPA as: the “care-autonomy” profile, “overprotection indifference” profile, “indifference” profile, and “undifferentiated parenting” profile. These results suggested that the risk of depression/anxiety symptoms among adolescents was lower in the “care-autonomy” profile, while the risk of depression was higher in the “indifference parenting” profile than in the “undifferentiated parenting” profile. A cross-sectional study (Eun et al., 2018) showed that different types of parenting styles were associated with depression/anxiety symptoms among adolescents, while “high maternal control” style was related to greater odds of depression/anxiety.

Simultaneously, this study found that resilience was related to depression/anxiety symptoms among adolescents. The resilience protection model suggested that resilience had a buffer effect on the negative effects of adversity on adolescents, and the higher the resilience score was, the lower the risk of negative consequences (Brookmeyer et al., 2005). It indicated that resilience may be a factor influencing depression/anxiety symptoms among adolescents. Skrove et al. (2013) demonstrated this, and also showed that resilience is a protective factor against adolescent depression/anxiety symptoms. A cohort study examined the impact of resilience on depression symptoms among left-behind children in China, and found that baseline resilience was related to follow-up depressive symptoms among children (Wu et al., 2017). These results were consistent with those of the current study.

At present, there are few studies on the relationship between parenting style, resilience, and depression/anxiety symptoms among adolescents. Similar studies have explored the correlation mediating effect of negative life events, resilience, and depressive symptoms in Chinese adolescents, and found that resilience was negatively associated with depression symptoms; among these, resilience partially mediated the impact of negative life events on depressive symptoms in Chinese adolescents (Liu et al., 2019). Anyan et al. (Anyan and Hjemdal, 2016) investigated the effect of stress on the relationship between resilience and anxiety/depression symptoms among adolescents. They found that resilience was inversely related to anxiety and depression symptoms, with resilience playing a partial mediating role between stress and anxiety/depression symptoms. In this study, we found no association between parenting style, resilience, depression, and anxiety symptoms among adolescents. The above studies indirectly suggest that different types of parenting styles may affect adolescent depression/anxiety symptoms through resilience, but given the limited research results, it remains to be seen whether the relevant theoretical hypotheses are valid and can be verified.

## Strength and Limitations

This study used more advanced statistical analysis (LPA) to identify the different patterns of parenting styles, and subsequently evaluate the relationship between patterns of parenting styles, resilience, and depression/anxiety symptoms among middle school students. The research ideas are novel and provide a reference for the promotion of adolescent mental health. However, this study has some limitations. First, this was a cross-sectional study; thus, we were unable to establish a causal relationship between variables, and cohort studies should be conducted to further explore these relationships. Second, the research object was limited to senior high school students in Hefei City. The sample size of the survey was small and extrapolation of the conclusions was limited. Future research with a larger cohort may wish to explore stability and transition patterns across parenting styles. Finally, our study questionnaire was completed subjectively by participants; thus, there may have been some recall bias. Therefore, combined with the EMBU child and parent versions (Mathieu et al., 2020), this study aimed to understand how depression/anxiety symptoms may be affected differently across these two reporting types.

## Conclusion

This study identified three profiles of parenting style practiced on Chinese adolescents using a LPA approach. Simultaneously, we investigated the influence of different parenting patterns and resilience on depression/anxiety symptoms among adolescents and their interaction effects. Future studies on exporting the risk and protective factors of depression/anxiety symptoms should consider the potential influence of different patterns of parenting styles and levels of resilience.

## Data Availability Statement

The raw data supporting the conclusions of this article will be made available by the authors, without undue reservation.

## Ethics Statement

The studies involving human participants were reviewed and approved by Ethics Committee of Anhui Medical University. Written informed consent to participate in this study was provided by the participants or their legal guardian/next of kin.

## Author Contributions

ZQ reviewed the topic related literature and drafted the first version of manuscript. ZQ and YG performed the study design, coordination, and data collection and worked on data analysis. JW involved in interpretation of the data and revision of the manuscript. HZ performed the study design and carried out study supervision and revision of the manuscript. All authors checked interpreted results and approved the final version.

## Conflict of Interest

The authors declare that the research was conducted in the absence of any commercial or financial relationships that could be construed as a potential conflict of interest.

## Publisher’s Note

All claims expressed in this article are solely those of the authors and do not necessarily represent those of their affiliated organizations, or those of the publisher, the editors and the reviewers. Any product that may be evaluated in this article, or claim that may be made by its manufacturer, is not guaranteed or endorsed by the publisher.
