# Supplementary material for: Associations of Parenting Style and Resilience With Depression and Anxiety Symptoms in Chinese Middle School Students
Source: Front Psychol. 2022 Jul 1;13:897339. doi: 10.3389/fpsyg.2022.897339 (PMC9285101; doi:10.3389/fpsyg.2022.897339)
Supplement: Supplementary file 1 [file Data_Sheet_1.docx]

**
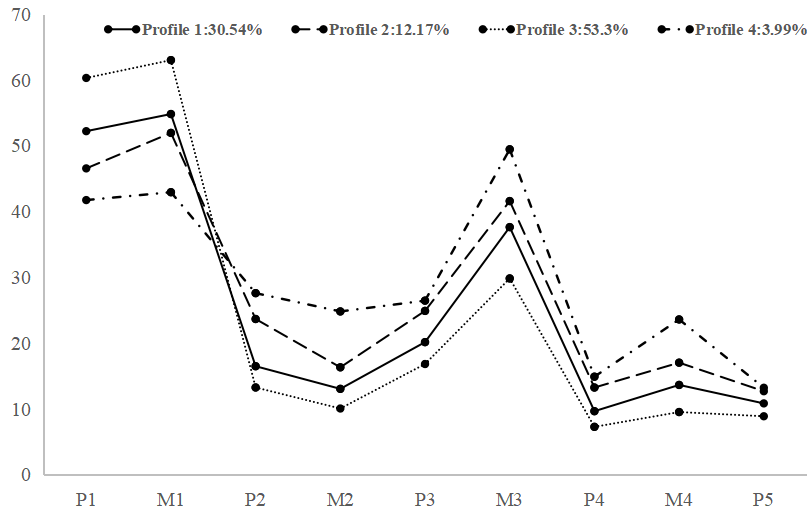
**

**Supplementary Figure 1 Plot of 4 latent profiles of parenting styles**

*(Note: P1=paternal emotional warmth; M1=maternal emotional warmth; P2=paternal severe punishment; M2=maternal severe punishment; P3=paternal excessive interference; M3=maternal excessive interference; P4=paternal rejection and denial; M4=maternal rejection and denial; P5=paternal over protection.)*

**Supplementary Table 1 Average posterior probabilities associated with the three-profile model.**

| Latent Profile | 1 | 2 | 3 |
| --- | --- | --- | --- |
| 1 | 0.966 | 0.034 | 0.000 |
| 2 | 0.055 | 0.929 | 0.016 |
| 3 | 0.000 | 0.046 | 0.954 |
| Note. Posterior probabilities are the probability that an individual belongs to the assigned profile and to no other profiles. | | | |

**Supplementary Table 2 Number, % and OR of depression symptoms by level of parenting style and resilience in girls and boys.**

| Variable | Girls | | |  | Boys | | |
| --- | --- | --- | --- | --- | --- | --- | --- |
|  | n(%) | *OR*(95%*CI*) ^b^ | *P* |  | n(%) | *OR*(95%*CI*) ^b^ | *P* |
| Parenting style |  |  |  |  |  |  |  |
| Positive parenting | 92(19.5) | 0.36(0.26-0.51) | <0.001 |  | 102(13.5) | 0.28(0.21-0.37) | <0.001 |
| Negative parenting | 31(64.6) | 2.71(1.42-5.16) | 0.002 |  | 67(47.2) | 1.60(1.09-2.35) | 0.017 |
| Moderate parenting | 97(40.2) | 1.00 |  |  | 152(35.8) | 1.00 |  |
| Resilience |  |  |  |  |  |  |  |
| Low | 209(36.5) | 9.95(5.25-18.86) | <0.001 |  | 295(30.2) | 5.54(3.60-8.51) | <0.001 |
| High | 11(5.9) | 1.00 |  |  | 26(7.6) | 1.00 |  |
| ^b^ Adjusted for only child, registered residence, self-reported academic performance, parents’ education level. | | | | | | | |

**Supplementary Table 3 Number, % and OR of anxiety symptoms by level of parenting style and resilience in girls and boys.**

| Variable | Girls | | |  | Boys | | |
| --- | --- | --- | --- | --- | --- | --- | --- |
|  | n(%) | *OR*(95%*CI*) ^b^ | *P* |  | n(%) | *OR*(95%*CI*) ^b^ | *P* |
| Parenting style |  |  |  |  |  |  |  |
| Positive parenting | 50(10.6) | 0.36(0.20-0.45) | <0.001 |  | 41(5.4) | 0.30(0.20-0.45) | <0.001 |
| Negative parenting | 23(47.9) | 1.85(1.17-2.91) | 0.008 |  | 37(26.1) | 1.85(1.17-2.91) | 0.008 |
| Moderate parenting | 60(24.9) | 1.00 |  |  | 68(16.0) | 1.00 |  |
| Resilience |  |  |  |  |  |  |  |
| Low | 117(20.5) | 2.70(1.61-4.52) | <0.001 |  | 128(13.1) | 2.87(1.65-5.02) | <0.001 |
| High | 16(8.5) | 1.00 |  |  | 18(5.3) | 1.00 |  |
| ^b^ Adjusted for only child, registered residence, self-reported academic performance, parents’ education level. | | | | | | | |

**Supplementary Table 4 Association between different level of resilience defined as ≥P_67_ and ≥P_90_ with depression/anxiety symptoms in adolescents.**

| Variable | Depression symptoms | |  | Anxiety symptoms | |
| --- | --- | --- | --- | --- | --- |
|  | Crude *OR* (95% *CI*) | Adjusted *OR* (95% *CI*)^a^ |  | Crude *OR* (95% *CI*) | Adjusted *OR* (95% *CI*)^a^ |
| P_67_ |  |  |  |  |  |
| Low | 5.76(4.32-7.67)^**^ | 6.12(4.57-8.20)^**^ |  | 2.91(2.09-4.05)^**^ | 3.03(2.16-4.24)^**^ |
| High | 1.00 | 1.00 |  | 1.00 | 1.00 |
| P_90_ |  |  |  |  |  |
| Low | 5.95(3.29-10.77)^**^ | 5.96(3.28-10.82)^**^ |  | 3.46(1.75-6.83)^**^ | 3.33(1.67-6.62)^**^ |
| High | 1.00 | 1.00 |  | 1.00 | 1.00 |
| ^a^ Adjusted for gender, only child, registered residence, self-reported academic performance, parents’ education level. ^**^*P* ≤ *0.001.* | | | | | |

**Supplementary Table 5 The prevalence of parenting style by** **level of resilience, n(%)**

| Parenting style | Resilience | | | Adjusted *OR*(95%*CI*) ^b^ | *P* |
| --- | --- | --- | --- | --- | --- |
|  | Low | High | *χ^2^* |  |  |
| Positive parenting | 834(68.1) | 390(31.9) | 64.178^**^ | 2.24(1.76-2.84) | ＜0.001 |
| Negative parenting | 163(85.8) | 27(14.2) |  | 0.81(0.51-1.28) | 0.361 |
| Moderate parenting | 552(83.0) | 113(17.0) |  | 1.00 |  |
| ^b^ Adjusted for gender, only child, registered residence, self-reported academic performance, parents’ education level. ^**^*P* < *0.001.* | | | | | |

**Table DS 6 Interaction effects between parenting style and resilience on depression and anxiety symptoms in adolescents.**

| Variable | Anxiety symptoms | |  | Depressive symptoms | |
| --- | --- | --- | --- | --- | --- |
|  | Crude *OR* (95% *CI*) | *P* |  | Crude *OR* (95% *CI*) | *P* |
| Parenting style |  |  |  |  |  |
| Positive parenting | 0.33(0.15-0.71) | 0.004 |  | 0.39(0.19-0.83) | 0.014 |
| Negative parenting | 1.75(0.57-5.41) | 0.333 |  | 1.75(0.57-5.41) | 0.333 |
| Moderate parenting | 1.00 |  |  | 1.00 |  |
| Resilience |  |  |  |  |  |
| Low | 2.74(1.89-3.98) | ＜0.001 |  | 6.43(4.53-9.13) | ＜0.001 |
| High | 1.00 |  |  | 1.00 |  |
| Parenting style×Resilience |  |  |  |  |  |
| Positive parenting×Low | 1.14(0.50-2.61) | 0.754 |  | 0.90(0.42-1.96) | 0.796 |
| Negative parenting×Low | 1.11(0.34-3.65) | 0.868 |  | 1.02(0.31-3.32) | 0.977 |
